# AI-Enhanced ECG Applications in Cardiology: Comprehensive Insights from the Current Literature with a Focus on COVID-19 and Multiple Cardiovascular Conditions

**DOI:** 10.3390/diagnostics14171839

**Published:** 2024-08-23

**Authors:** Luiza Camelia Nechita, Aurel Nechita, Andreea Elena Voipan, Daniel Voipan, Mihaela Debita, Ana Fulga, Iuliu Fulga, Carmina Liana Musat

**Affiliations:** 1 Faculty of Medicine and Pharmacy, Dunarea de Jos University of Galati, 800008 Galati, Romania; luiza.nechita@ugal.ro (L.C.N.); aurel.nechita@ugal.ro (A.N.); mihaela.debita@ugal.ro (M.D.); ana.fulga@ugal.ro (A.F.); iuliu.fulga@ugal.ro (I.F.); carmina.musat@ugal.ro (C.L.M.); 2 Faculty of Automation, Computers, Electrical Engineering and Electronics, Dunarea de Jos University of Galati, 800008 Galati, Romania

**Keywords:** artificial intelligence, electrocardiography, cardiology, COVID-19, cardio-oncology, machine learning (ML), deep learning (DL), convolutional neural networks (CNNs), diagnosis, risk prediction

## Abstract

The application of artificial intelligence (AI) in electrocardiography is revolutionizing cardiology and providing essential insights into the consequences of the COVID-19 pandemic. This comprehensive review explores AI-enhanced ECG (AI-ECG) applications in risk prediction and diagnosis of heart diseases, with a dedicated chapter on COVID-19-related complications. Introductory concepts on AI and machine learning (ML) are explained to provide a foundational understanding for those seeking knowledge, supported by examples from the literature and current practices. We analyze AI and ML methods for arrhythmias, heart failure, pulmonary hypertension, mortality prediction, cardiomyopathy, mitral regurgitation, hypertension, pulmonary embolism, and myocardial infarction, comparing their effectiveness from both medical and AI perspectives. Special emphasis is placed on AI applications in COVID-19 and cardiology, including detailed comparisons of different methods, identifying the most suitable AI approaches for specific medical applications and analyzing their strengths, weaknesses, accuracy, clinical relevance, and key findings. Additionally, we explore AI’s role in the emerging field of cardio-oncology, particularly in managing chemotherapy-induced cardiotoxicity and detecting cardiac masses. This comprehensive review serves as both an insightful guide and a call to action for further research and collaboration in the integration of AI in cardiology, aiming to enhance precision medicine and optimize clinical decision-making.

## 1. Introduction

Artificial intelligence (AI) is being increasingly explored for its applications in electrocardiography to aid in diagnosis, classification, and patient management. Analyses of AI-enhanced electrocardiograms (AI-ECGs) can greatly improve the accuracy of diagnoses as well as the overall management and treatment of patients’ health conditions [1]. The use of AI in ECG interpretation showcases its transformative effect on cardiovascular medicine, allowing for swift and accurate analysis and detection of various conditions, with significant benefits for both individual patient care and broader public health outcomes due to advancements in digital health technologies [2].

By using deep learning (DL) techniques, which involve training neural networks (NN) to mimic the human brain’s ability to learn from data, AI can analyze ECGs, leading to fully automated models. These AI algorithms act as non-invasive biomarkers, aiding in the identification of subtle patterns and signals in ECGs that might be difficult for human interpreters to detect [3]. Integrating AI with ECG represents a major advancement in cardiovascular medicine, significantly augmenting the capabilities of traditional ECGs used for diagnosing heart diseases. AI-ECG excels at identifying subtle changes and intricate patterns in cardiac waveforms, achieving a level of precision and sensitivity that conventional methods could not previously reach [4].

There is a growing trend to enhance the accuracy and scalability of automated ECG analysis, driven by the availability of extensive digital ECG data and the application of DL algorithms. This relatively new approach, already under exploration in numerous studies and research, represents a significant opportunity to advance the field [5]. Furthermore, it opens new doors for future collaboration between AI and the medical field of diagnostics, potentially making its integration a necessity.

A novel challenge in the medical field has been the emergence of COVID-19, which AI has effectively addressed. When COVID-19 intersects with cardiac issues, the demand for AI applications becomes even more pronounced. The application of AI-ECG has shown potential in the early prediction of COVID-19 severity, leading to improved patient care strategies [6]. As the literature already shows, adverse events associated with COVID-19 can be anticipated using AI-ECG. AI can detect subtle signs of myocardial involvement in the 12-lead ECG, thereby predicting complications [7].

**Figure 1:**
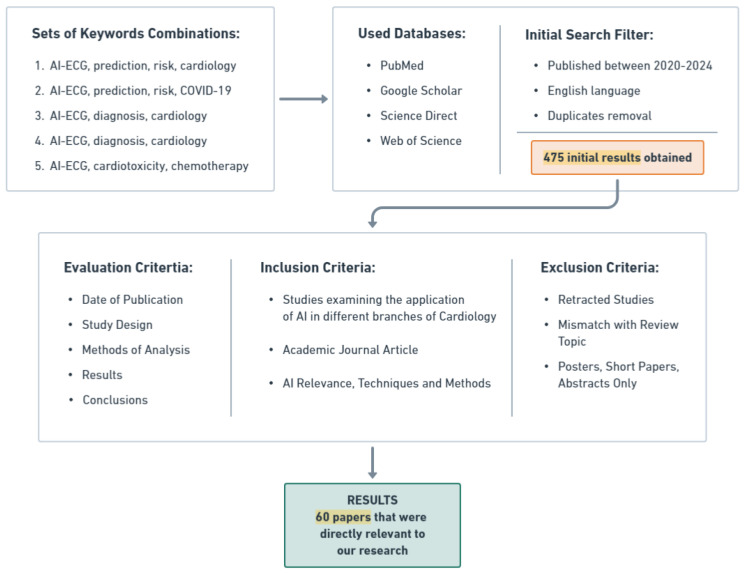
Systematic literature review process for AI-ECG applications in cardiology and COVID-19.

Our search strategy for identifying the relevant literature on AI-ECG in cardiology and COVID-19 involved a systematic approach, as illustrated in Figure 1. We performed a comprehensive review of the current literature, including original articles on various clinical applications of AI-ECG in cardiology and the COVID-19 pandemic. Extensive searches were conducted across PubMed, Google Scholar, Science Direct, and Web of Science databases to identify relevant manuscripts. We used five sets of keywords including AI-ECG, prediction, risk, cardiology, diagnosis, COVID-19, and cardiotoxicity. 

We limited our search to papers published in English between 2020 and 2024, yielding 475 relevant publications. After removing duplicates, we assessed the abstracts and titles for inclusion based on the application of AI-ECG in cardiovascular disease and COVID-19 infection. Selection criteria included journal quality, date of publication, study design, analysis methods, results, AI methods, and conclusions. 

After a thorough review and assessment, we identified and included 60 studies directly relevant to our research, providing valuable insights into the use and impact of AI-ECG in cardiology and infectious diseases, particularly during the COVID-19 pandemic. These studies will be depicted, explained, compared, and categorized by disease throughout this article. The subsequent sections will introduce key concepts from AI and neural networks (NN), illustrated through a review of the literature on cardiovascular diseases, with a focus on AI-ECG applications in cardiac symptoms associated with COVID-19. This comprehensive examination will reveal trends in NN over the past 5 years and highlight methods demonstrating superior performance in the addressed cases. Additionally, this review includes a section on using AI-ECG as a diagnostic tool for detecting cardiotoxicity from chemotherapy and evaluating cardiac masses.

## 2. Basic Concepts of Artificial Intelligence and Machine Learning for Healthcare

### 2.1. Introduction to AI

Artificial intelligence (AI) refers to the simulation of human intelligence processes by machines, particularly computer systems. These processes include learning (the acquisition of information and rules for using the information), reasoning (using rules to reach approximate or definite conclusions), and self-correction [8]. In healthcare, AI aims to mimic cognitive functions such as problem-solving and decision-making in clinical environments. The integration of AI into healthcare systems promises enhanced diagnostic accuracy, personalized treatment plans, and optimized patient outcomes [9].

AI can be categorized into different types based on capabilities and functionalities:

- Narrow AI (Weak AI): Designed to perform a narrow task, such as facial recognition or internet searches. It operates under a limited set of constraints and cannot perform tasks outside its specific domain.
- General AI (Strong AI): Possesses the ability to understand, learn, and apply knowledge in a way that is indistinguishable from human intelligence. General AI remains largely theoretical and is not yet realized [10].
- Artificial Superintelligence (AS): An intelligence that surpasses human intelligence in all aspects, including creativity, general wisdom, and problem-solving. This type is also theoretical and a topic of debate and research in AI ethics and safety [11].
- In the medical field, narrow AI is primarily used to assist with tasks such as diagnostic imaging, patient data analysis, and personalized treatment recommendations due to its ability to perform specific, well-defined tasks with high accuracy and efficiency. General AI is still in the developmental stage and AS is a topic of ongoing debate and research in AI ethics and safety.

AI relies on several core techniques to mimic cognitive functions:

- Machine learning (ML): Involves training algorithms to learn from data and make predictions or decisions without being explicitly programmed [12].
- Deep learning (DL): Uses neural networks (NN) with many layers (deep networks) to model complex patterns in data. DL has been particularly successful in image and speech recognition tasks [13].
- Natural language processing (NLP): Enables machines to understand and interact using human language. Applications include language translation, sentiment analysis, and chatbots [13].
- Robotics: Combines AI with mechanical engineering to create robots capable of performing tasks autonomously or semi-autonomously, often in complex and dynamic environments [12].

**Figure 2:**
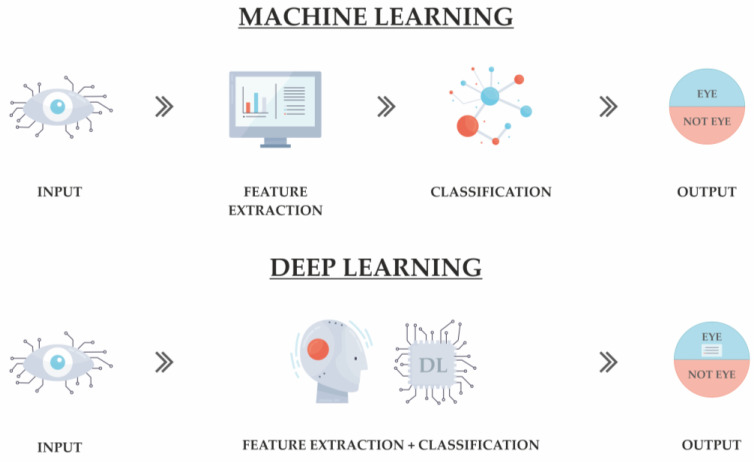
Comparison of traditional machine learning and deep learning approaches.

Even though DL is a subset of ML, it sets itself apart using neural networks with multiple layers, enabling the modeling of intricate data patterns. NN are inspired by the structure of the human brain. They comprise layers of interconnected nodes (neurons) that collaboratively process information and learn from data [14]. These networks excel in pattern recognition and predictive tasks due to their ability to adapt and improve over time. Figure 2 illustrates the differences between traditional ML and DL approaches:

While AI encompasses a broad range of techniques and applications, one of the most significant and widely used subsets is machine learning. ML plays a crucial role in the development of AI applications, especially in healthcare, where it drives innovations in diagnostics, treatment planning, and patient management. In the next section, we will delve deeper into the fundamentals of ML, its methodologies, and its transformative impact on healthcare.

### 2.2. Introduction to Machine Learning

ML entails creating algorithms that can analyze data, learn from it, and make informed predictions or decisions autonomously. It includes the following subtypes:

- Supervised learning: This approach uses labeled data to train the algorithm. Each input comes with a corresponding output label, which helps the algorithm learn the relationship between inputs and outputs [15]. It is commonly used in predictive analytics, such as forecasting heart disease from patient data.
- Unsupervised learning: In this method, the algorithm is given data without explicit instructions on what to do with them [8]. It must find patterns and relationships within the data on its own. This technique is often used for clustering patients based on symptoms or for identifying unknown correlations in medical data.
- Semi-supervised learning: Combines both labeled and unlabeled data during training. This method is useful when acquiring a fully labeled dataset is impractical or expensive [8]. It leverages the small amount of labeled data to better understand and label the large amount of unlabeled data.
- Reinforcement learning: This technique involves training algorithms to make a sequence of decisions by rewarding them for desirable behaviors and penalizing them for undesirable ones. It is particularly useful in optimizing treatment protocols where the algorithm learns the best strategies through trial and error [15].

**Figure 3:**
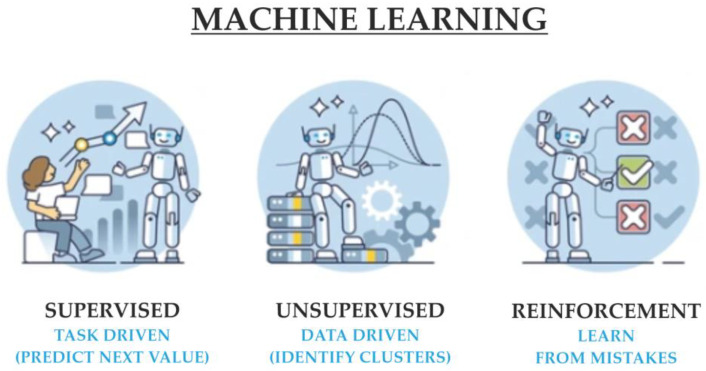
Overview of supervised, unsupervised, and reinforcement ML techniques.

The Figure 3 below illustrates these types:

#### Description of ML Algorithms Explored in This Article

Because this paper addresses studies where various algorithms are used, to keep the information about AI and ML algorithms concise, Table 1 briefly describes the ones that are relevant and encountered in the studies that will be discussed further.

## 3. AI and ML in Healthcare and Cardiology: AI-ECG for Heart Diseases

AI and ML are transforming the field of cardiology by enhancing the diagnosis, treatment, and risk prediction of cardiovascular diseases. This section delves into several key areas where AI-ECG is making significant strides. The research papers relevant to these applications and findings of AI and ML methods in risk prediction for heart diseases are summarized in Table 2, Section 3.1, and those focusing on diagnosis are detailed in Table 3, specifically in Section 3.2, showcasing the advancements in identifying high-risk patients and improving diagnostic accuracy.

### 3.1. Risk Prediction in Heart Diseases

#### 3.1.1. Risk Prediction of Arrhythmias

Arrhythmias are disturbances in the heart’s electrical conduction system, leading to abnormal heart rhythms that can manifest as tachycardia, bradycardia, or irregular contractions. These deviations from the normal cardiac rhythm can result in compromised hemodynamic function and are associated with an increased risk of adverse cardiovascular events, including stroke and heart failure. Atrial fibrillation (AF) is one of the most common and serious arrhythmias, often resulting in stroke and heart failure. AI and ML have been effective in identifying and predicting AF, improving early diagnosis and treatment.

A review of the studies summarized in Table 2 reveals that CNN and DL models are particularly effective in the prediction and diagnosis of AF. These models excel in analyzing complex ECG data and detecting subtle patterns, providing higher accuracy than traditional ML methods. The studies demonstrate that these advanced algorithms significantly improve early diagnosis and treatment planning for AF. However, they require large datasets for training and are computationally intensive, which can be a limitation in resource-constrained settings. Notably, cited papers [17,18,19,20,21,22,23,24,25,26] provide detailed evidence of the efficacy of AI-ECG methods in identifying high-risk features for AF, predicting AF from normal sinus rhythms, and evaluating the likelihood of AF recurrence following catheter ablation.

#### 3.1.2. Heart Failure

Heart failure (HF) is a chronic condition where the heart cannot pump blood efficiently, leading to symptoms like shortness of breath, fatigue, and fluid retention. AI significantly contributes to predicting HF using ECG signals, enabling early intervention to reduce morbidity and mortality. For instance, Grun D. [27] demonstrates that AI can predict HF with high accuracy using standard 12-lead ECG signals, while Akbilgic O. [28] highlights the role of AI in aiding early intervention, which helps mitigate severe outcomes of HF by identifying patients at risk earlier, allowing for timely and targeted treatment plans.

CNNs are particularly suited for predicting HF due to their ability to capture complex patterns and structures in ECG signals. They excel in recognizing spatial patterns within the data, which is critical for accurately predicting HF. CNNs can handle the high dimensionality of ECG data, extracting relevant features without extensive pre-processing. Other ML methods, such as decision trees or support vector machines, generally require more manual feature extraction and may not achieve the same level of accuracy due to their limitations in handling complex, high-dimensional data.

Overall, CNN models provide a promising tool for predicting HF, significantly enhancing the ability to intervene early and manage the condition more effectively. These models demonstrate the potential for AI to transform HF prediction and treatment, improving patient outcomes through advanced data analysis and timely clinical interventions.

#### 3.1.3. Pulmonary Hypertension

Pulmonary hypertension (PH) is a serious condition characterized by high blood pressure in the arteries that carry blood from the heart to the lungs. Early diagnosis is crucial for managing disease progression and preventing mortality.

Kwon J.M. [29] demonstrated that an AI algorithm using DL methods shows high accuracy in predicting PH with both 12-lead and single-lead ECGs, significantly improving early diagnosis and patient outcomes. A 12-lead ECG provides a comprehensive view of the heart’s electrical activity from multiple angles, while a single-lead ECG captures the heart’s rhythm from one perspective, often used for continuous monitoring.

DL is highly effective for PH prediction due to its ability to handle complex, multi-dimensional data. It captures intricate patterns within ECG signals, which is essential for accurate early diagnosis. Unlike traditional machine learning methods that require manual feature extraction and struggle with high-dimensional data, DL models excel in processing large datasets and identifying subtle patterns indicative of PH. This high level of accuracy and reliability in complex data analysis makes DL the preferred choice for PH prediction, enhancing early intervention and improving patient outcomes.

#### 3.1.4. Mortality Prediction

Predicting mortality and sudden cardiac death (SCD) is crucial in cardiovascular disease management. AI and DL models significantly enhance prediction by analyzing ECG data. Tsai D.J. [30] demonstrates that a DL-based survival model establishes critical values from ECG data, predicting SCD and enhancing cardiovascular disease management. Similarly, Baek Y.S. [31] shows how DL models estimate cardiac age and predict mortality using 12-lead ECGs, providing superior accuracy in mortality prediction.

DL models have proven to be highly effective in identifying cases at risk of SCD with improved accuracy over conventional ECG risk models. They establish critical values from ECG data, exploring associations with cardiovascular events, thus enhancing prediction and stratification of SCD risk among HF patients. Additionally, Liu C.M. [33] highlights that AI models can identify patients with pulmonary hypertension (PH) and predict their risk of cardiovascular mortality, enabling early intervention.

As shown in studies like Shiraishi Y. [32], both DL and CNN models have been employed in predicting mortality and SCD. However, DL models generally provide superior performance in mortality prediction due to their ability to handle complex datasets and offer nuanced risk stratification. These models excel in analyzing intricate ECG data to predict mortality and SCD with high accuracy, significantly improving early diagnosis and intervention strategies. This capability makes them more reliable than CNN models, which, while effective, may not achieve the same level of accuracy due to their limitations in handling complex data structures. This makes DL models the preferred choice for enhancing early intervention and improving patient outcomes in the context of mortality prediction and management of cardiovascular diseases.

#### 3.1.5. Cardiomyopathy

Cardiomyopathy refers to diseases of the heart muscle that can lead to HF and other complications. Güntürkün F. [34] demonstrated that AI-driven analysis of baseline ECGs can predict the risk of late-onset cardiomyopathy in childhood cancer survivors, enabling preventive interventions and personalized monitoring.

Additionally, Siontis K.C. [2] highlights that DL models can detect hypertrophic cardiomyopathy (HCM) with high accuracy using standard 12-lead ECGs, improving early identification and management.

Furthermore, Ko W.Y. [35] underscores the effectiveness of CNN models in the detection of HCM, complementing traditional diagnostic methods by enhancing both speed and accuracy. CNNs, with their superior ability to analyze complex and high-dimensional ECG data, are particularly well suited for identifying structural heart abnormalities, which is crucial for the early diagnosis and management of cardiomyopathy. However, these models require comprehensive datasets for training and may benefit from integration with additional clinical data to optimize their predictive accuracy.

Overall, CNN models provide a reliable and accurate tool for predicting and diagnosing cardiomyopathy, significantly enhancing early intervention and improving patient outcomes. These models highlight the transformative potential of AI in managing and treating complex cardiovascular conditions.

#### 3.1.6. Chemotherapy Cardiotoxicity (Cardio-Oncology)

Chemotherapy-induced cardiotoxicity is a critical concern in oncology, particularly with the use of anthracyclines and trastuzumab, which can cause significant heart problems such as left ventricular dysfunction and heart failure. Early detection and intervention are vital to prevent permanent damage and ensure the continuation of cancer therapy.

Jacobs JEJ. [36] (2024) demonstrated that AI-enhanced ECG (AI-ECG) can identify early changes in left ventricular ejection fraction in patients undergoing anthracycline therapy, enabling timely interventions. Similarly, Yagi R. [37] (2024) showed that AI-driven analysis of baseline ECGs could predict the risk of cardiotoxicity in chemotherapy patients, offering a non-invasive approach to monitoring cardiac health.

AI models, particularly those utilizing CNNs and DL, are proving highly effective in predicting and diagnosing chemotherapy-induced cardiotoxicity. These models enhance early identification, which is crucial for preventing long-term cardiac damage and improving patient outcomes. Furthermore, Halasz G. [38] (2024) emphasized the importance of AI-assisted ECG in diagnosing cardiac dysfunction related to cancer therapy, particularly in resource-limited settings. This highlights the accessibility and transformative potential of AI in global healthcare.

Moreover, the work by Güntürkün F. [34] (2021) on childhood cancer survivors underscores the potential of AI in predicting late-onset cardiomyopathy, enabling continuous monitoring and timely interventions for at-risk patients long after cancer treatment.

Overall, these AI-driven tools significantly improve the early detection and management of chemotherapy-induced cardiotoxicity, ultimately leading to better patient outcomes and enhanced quality of life for cancer survivors.

### 3.2. Diagnosis in Heart Diseases

#### 3.2.1. Diagnosis of Arrhythmias

Diagnosis and risk prediction for arrhythmias serve complementary roles in cardiovascular care. Diagnosis focuses on identifying arrhythmias at the time of examination using tools like ECGs. AI and DL models analyze ECG data in real-time or from past records to detect conditions such as atrial fibrillation (AF), ventricular tachyarrhythmias (VTA), and long QT syndrome, enabling prompt treatment. Key aspects of AI-enhanced ECG diagnosis include real-time detection of abnormal heart rhythms, confirmation of arrhythmias not apparent through traditional methods, and quick decision-making for immediate treatment interventions.

Risk prediction assesses the likelihood of future arrhythmias using historical data and predictive models. This approach identifies high-risk individuals for preventive measures and closer monitoring. AI-enhanced risk prediction allows for identifying individuals at high risk before symptoms appear, enabling targeted preventive interventions and informing long-term management strategies to mitigate potential complications.

Both approaches are crucial for comprehensive cardiovascular disease management, with AI and DL models significantly enhancing accuracy and effectiveness. Baek Y.S. [39] demonstrates that a DL-based algorithm shows high accuracy in detecting AF from 12-lead ECGs during normal sinus rhythm, and in [40] confirms the efficacy of DL models in clinical settings for AF detection.

After analyzing the studies summarized in, DL models are preferred for diagnosis of arrhythmias due to their superior performance in detecting complex and subtle heart rhythm patterns. For instance, Sabut S. [41] highlights the effectiveness of AI in detecting ventricular tachyarrhythmia conditions, while Bos J.M. [42] shows AI’s ability to differentiate patients with electrocardiographically concealed long QT syndrome.

DL models generally offer superior performance compared to DNNs in diagnosing arrhythmias due to their capability to process and analyze high-dimensional data. While DNNs are effective, DL models, which often incorporate deeper layers and convolutional networks, excel in capturing intricate details and patterns within ECG data.

In conclusion, while both DL and DNN models are effective in diagnosing arrhythmias, DL models are preferred for their superior ability to handle complex and high-dimensional data, leading to higher accuracy in detection and better patient outcomes.

#### 3.2.2. Mitral Regurgitation

Mitral regurgitation (MR) is a heart condition where the mitral valve does not close properly, allowing blood to flow backward into the left atrium from the left ventricle (LV). Early diagnosis is essential to prevent the irreversible progression of this condition. Kwon J.M. [43] demonstrated that AI algorithms show promising results in detecting MR using both 12-lead and single-lead ECGs, highlighting the potential of AI in improving diagnosis and treatment of this condition.

DL models have proven highly effective in the early diagnosis of MR by processing complex ECG data to detect subtle anomalies indicative of mitral valve dysfunction. This leads to more accurate and earlier diagnoses compared to traditional methods, significantly improving the management of MR. Although DL models require large datasets for training and are computationally intensive, the benefits of timely intervention and better management of MR outweigh these challenges.

Overall, DL models offer a reliable and accurate tool for diagnosing mitral regurgitation, significantly enhancing early intervention and patient care. Despite the need for substantial data and computational resources, their transformative potential in managing complex cardiovascular conditions is evident.

#### 3.2.3. Hypertension

Hypertension, or high blood pressure (HBP), is a major risk factor for heart disease, stroke, and kidney disease. Early and accurate detection is crucial for effective management. Soh D. C.K. [44] proposed an AI tool using ECG signals for detecting HBP and identifying masked hypertension (MHPT), which enables automatic classification of HPT signals compared to normal ECG signals. This tool is particularly useful for hospital diagnoses and continuous monitoring.

KNN models, as shown in [44] are suitable for detecting hypertension due to their simplicity and effectiveness in small-scale studies. These models provide an efficient tool for early detection, allowing for the timely and accurate classification of ECG signals in hospital settings where rapid diagnosis is essential.

However, KNN models are less effective for large datasets and may struggle with handling complex relationships, which limits their application in more extensive studies. Despite these limitations, KNN models offer a valuable tool for detecting hypertension, especially in settings where quick and straightforward analysis is required.

#### 3.2.4. Pulmonary Embolism

Pulmonary embolism (PE) is a serious condition characterized by the obstruction of a pulmonary artery, usually caused by a thrombus migrating from a peripheral vein. Early and accurate diagnosis is essential to reduce mortality and morbidity.

In contrast to risk prediction, which aims to assess the likelihood of developing PE in the future, diagnosis focuses on the immediate detection of the condition. Early diagnosis of PE allows for prompt treatment, which is critical in emergency settings to reduce mortality and morbidity. A DL-based AI model for PE diagnosis using 12-lead ECGs developed by Valente S. B [45] has demonstrated high specificity, showcasing AI’s potential in emergency medical diagnostics.

DNNs are effective for diagnosing PE due to their ability to model complex relationships in clinical data. These models provide high specificity in diagnostics where timely and accurate identification of PE is crucial.

Overall, DNN models offer a robust solution for diagnosing PE, enhancing the ability of healthcare providers to quickly and accurately identify this serious condition and improve patient outcomes. They provide high specificity in diagnosis, making them extremely useful in emergency medical diagnostics. However, they require large datasets for training and are computationally intensive.

#### 3.2.5. Myocardial Infarction

Myocardial infarction (MI) and acute myocardial infarction (AMI) are serious heart conditions that require timely diagnosis to reduce morbidity and mortality. AI and DL have shown significant promise in enhancing the diagnosis of MI and AMI.

Liu W.C. [46,47] demonstrated successful detection capabilities for MI and AMI with both 12-lead and 6-lead ECG devices, improving diagnostic processes across different configurations. Additionally, the AI-S trial by Zhao Y. [49] demonstrated exceptional diagnostic capability for ST-segment elevation acute myocardial infarction (STEMI), significantly reducing the time from presentation to treatment in emergency settings. Cho Y. [48] (2020) also highlights the effectiveness of CNN models in detecting myocardial infarction, specifically in identifying STEMI cases.

DL and CNN models are both highly effective for myocardial infarction detection. DL models offer advantages in modeling complex data relationships, which can be crucial for accurate diagnosis in emergency settings, while CNNs also provide high accuracy in detecting MI and AMI, making them useful tools for timely intervention. While these models require large datasets for training and are computationally intensive, their high accuracy and utility in emergency settings make them invaluable for the early diagnosis and treatment of myocardial infarction.

Overall, both DL and CNN models significantly enhance the ability to diagnose myocardial infarction, improving patient outcomes through timely and accurate detection.

#### 3.2.6. Cardiac Masses in Cardio-Oncology

Cardiac masses in oncology patients pose a significant diagnostic challenge, as differentiating them from non-cancerous conditions can be difficult. Accurate and timely diagnosis is essential for guiding appropriate treatment and management.

Oikonomou E.K. [50] demonstrated that CNN-based models can effectively stratify cancer-related cardiac dysfunction using ECG imaging, enhancing the identification and characterization of cardiac masses. Similarly, Martinez DS [2] (2022) highlighted the application of deep learning (DL) models to improve ECG-based diagnosis of cardiac masses, particularly in complex cases where traditional methods may be insufficient.

Martinez D.S. [51] emphasized the importance of AI-enhanced ECG in improving diagnostic accuracy, especially in challenging cases where traditional methods may fall short. Meanwhile, Oikonomou EK [1] (2024) illustrated the role of AI in stratifying cardiac dysfunction related to cancer therapy, highlighting AI’s potential in both diagnosis and risk prediction.

While AI-ECG models require significant computational resources and large datasets for training, their ability to analyze complex data relationships and provide accurate diagnoses makes them invaluable in clinical settings. These tools enable earlier and more precise identification of cardiac masses, allowing for timely intervention and improved patient outcomes.

### 3.3. Chapter Summary and Conclusions

In this chapter, we focused on explaining several heart diseases and the applications of AI for risk prediction and diagnosis from a medical perspective. Our review covered the use of AI-ECG and various ML algorithms in the context of arrhythmias HF, PH, mortality prediction, cardiomyopathy, mitral regurgitation, hypertension, PE, MI, and cardio-oncology.

DL models have shown high accuracy in diagnosing arrhythmias, myocardial infarction, and mitral regurgitation, significantly improving early intervention and patient outcomes. CNNs have demonstrated their proficiency in predicting and diagnosing cardiomyopathy, particularly in high-risk groups such as childhood cancer survivors. These advanced algorithms’ ability to process and analyze large datasets allows them to identify subtle patterns and anomalies that traditional methods might miss.

Meanwhile, simpler models like traditional ML and KNN retain their value for specific applications, especially where computational resources or data availability are constrained. KNN, for instance, has been effectively used in detecting hypertension, providing a practical and reliable tool for hospital diagnoses and continuous monitoring.

The research and application of AI in cardiology highlight the potential for these technologies to further advance precision medicine, improve patient outcomes, and enhance clinical decision-making. As research continues to evolve, we can expect AI to play an increasingly integral role in the management and treatment of cardiovascular diseases, offering new opportunities for early diagnosis, risk prediction, and personalized care. The ongoing development and refinement of AI algorithms will likely lead to even more sophisticated tools and techniques, driving further improvements in cardiovascular health care.

## 4. AI Applications in COVID-19 and Cardiology

### 4.1. Impact of COVID-19 in Cardiovascular Health

The COVID-19 pandemic has significantly impacted cardiovascular health, presenting a range of challenges and consequences for both patients and healthcare systems. The overwhelming influx of COVID-19 patients placed unprecedented strain on healthcare resources, diverting attention and resources away from routine cardiovascular care. Hospitals faced critical shortages of beds, medical equipment, and staff, leading to the postponement of elective procedures and routine cardiovascular assessments.

Due to the focus on managing COVID-19 cases, many non-urgent medical appointments and procedures were delayed or canceled. This disruption resulted in delayed diagnoses and treatments for cardiovascular conditions such as hypertension, HF, and coronary artery disease. The deferral of elective surgeries and routine check-ups increased the risk of complications and adverse outcomes for cardiovascular patients.

The pandemic induced several indirect factors that exacerbated cardiovascular risks [52]:

- Lifestyle changes: Lockdowns and social distancing measures led to reduced physical activity, poor diet, and increased stress, all of which are detrimental to cardiovascular health.
- Mental health: The psychological impacts of the pandemic, including anxiety and depression, further contributed to cardiovascular risks.
- COVID-19 infection: The virus itself has been shown to have direct cardiovascular effects, including myocarditis, arrhythmias, and thromboembolic events. These complications posed significant risks, particularly for patients with pre-existing cardiovascular conditions.

Patients with chronic cardiovascular conditions faced significant challenges in managing their health during the pandemic. Access to regular monitoring and medication management was hindered by lockdowns and the redirection of healthcare resources. This disruption in routine care led to poorer control of chronic conditions and increased the likelihood of acute cardiovascular events.

The pandemic underscored the need for extensive research into the cardiovascular implications of COVID-19. Studies focused on understanding the mechanisms by which the virus affects the heart and blood vessels, leading to improved treatment protocols and preventive measures for at-risk populations. This research is crucial for developing strategies to mitigate the long-term cardiovascular impacts of the pandemic.

The COVID-19 pandemic has profoundly affected cardiovascular health, highlighting the vulnerabilities in managing chronic conditions during a global health crisis. While the challenges were significant, the pandemic also accelerated the adoption of telemedicine and emphasized the importance of resilient healthcare systems. Moving forward, these insights will be essential for enhancing cardiovascular care and preparing for future public health emergencies.

### 4.2. AI in COVID-19: Role of Diagnosis, Monitoring and Risk Assessment

AI has been instrumental in diagnosing COVID-19 by analyzing medical imaging (such as chest X-rays and CT scans), detecting viral RNA sequences in PCR tests, and evaluating symptoms through ML algorithms. These tools have enabled quicker and more accurate identification of COVID-19 cases, facilitating timely isolation and treatment. It was utilized for continuous analysis of patient data, including vital signs and oxygen levels. This real-time monitoring allowed healthcare providers to detect early signs of deterioration, adjust treatments, and predict outcomes, thereby improving patient management and resource allocation [53].

AI algorithms evaluated an individual’s likelihood of contracting COVID-19, experiencing severe disease, or developing complications by analyzing demographic data, pre-existing conditions, genetic markers, and lifestyle factors. This helped healthcare systems prioritize high-risk individuals for testing and vaccination, and tailor preventive strategies.

Early and accurate COVID-19 diagnosis using AI identified patients at higher risk of cardiovascular complications, such as myocarditis or thromboembolic events, allowing for early intervention. AI tools detected early signs of cardiovascular stress in COVID-19 patients, enabling timely interventions to prevent severe cardiovascular events, especially in those with pre-existing conditions. Their models identified individuals with underlying cardiovascular issues who were at higher risk of severe COVID-19 outcomes. Prioritizing these individuals for preventive measures reduced severe cardiovascular complications.

The management of COVID-19 was efficiently enhanced by AI through improved diagnosis, monitoring, and risk assessment. By linking these advancements to cardiovascular health, AI helped manage the immediate impacts of COVID-19 and mitigated long-term cardiovascular risks. This integrated approach ensured comprehensive care for patients, optimizing outcomes during and beyond the pandemic.

### 4.3. Risk Prediction in the COVID-19 Pandemic

The COVID-19 pandemic significantly impacted patients with cardiovascular diseases, making risk prediction essential for improving outcomes. AI-ECG has shown promise in predicting the severity of COVID-19 and associated complications in these patients, highlighting its potential as a critical tool in clinical decision-making.

#### 4.3.1. Complication Prediction Associated with COVID-19

AI-ECG allows for early determination of COVID-19 severity, aiding in patient management and resource allocation. Studies have shown that AI can predict the severity of COVID-19 in hospitalized patients, improving patient management [7,54,55]. Efficient-ECGNet has demonstrated robustness in classifying COVID-19 samples based on ECGs, highlighting AI’s potential in this domain. Additionally, AI helps identify subtle signs of myocardial involvement in the 12-lead ECG and can predict complications, which are crucial for protecting healthcare workers (HCWs) and patients.

To further enhance this section, we have conducted an analysis of studies that utilized various ML algorithms to predict complications associated with COVID-19 in cardiology. Table 4 provides a detailed comparison of the algorithms used, their strengths and weaknesses, accuracy, clinical relevance, data sources, and key findings. This analysis helps in identifying the most effective methods for predicting cardiovascular complications in COVID-19 patients.

From this analysis, we recommend a hybrid approach combining the temporal pattern recognition capabilities of CNN-LSTM with the interpretability and robustness of the extra tree classifier for the best prediction of complications in COVID-19 cardiology patients. This hybrid approach leverages the strengths of both models, providing a comprehensive tool for clinical decision-making.

#### 4.3.2. Mortality Risk Prediction

AI-ECG is also valuable in predicting mortality risk in patients with COVID-19. Neural networks and ECG-based prediction models have been shown to be effective in risk stratification. For example, DL models developed using advanced repeated structuring and learning procedures (AdvRS&LP) assist medical personnel in interpreting ECG patterns associated with various pathologies, aiding in early intervention and treatment [56].

Table 5 compares two studies focusing on mortality prediction in COVID-19 patients using electrocardiographic data and ML algorithms. Sbrollini et al. used the AdvRS&LP combined with LIME for interpretability, achieving a high AUC and clinical relevance due to the interpretability of the model. Van de Leur et al. [57] compared logistic regression, LASSO, and a pre-trained DNN, showing that the DNN model performed well but had limited added value over simple clinical variables and showed decreased performance during external validation. Based on the comparison, Sbrollini et al. [56]s’ approach is more robust and clinically relevant due to its higher accuracy and interpretability.

From this analysis, a hybrid approach combining the interpretability of AdvRS&LP with LIME and the robustness of the DNN model is recommended for the best prediction of mortality risk in COVID-19 patients. This hybrid approach leverages the strengths of both models, providing a comprehensive tool for clinical decision-making.

### 4.4. Diagnosis in the COVID-19 Pandemic

#### 4.4.1. COVID-19 Diagnosis Using AI-ECG

The COVID-19 pandemic has driven significant advancements in medical diagnostics, particularly through the application of AI and ML techniques. These innovations have proven essential in enhancing the accuracy and speed of diagnosing COVID-19, using tools such as ECG signals to provide non-invasive and rapid diagnostic methods. AI algorithms, especially those leveraging DL, have shown high specificity in diagnosing COVID-19. These models can detect specific changes in ECG signals caused by the virus, presenting a promising non-invasive diagnostic approach crucial during the pandemic. Table 6 below compares 12 studies where AI and DL models used for diagnosing COVID-19 are depicted and compared.

Based on the review of 12 studies focusing on CNNs for COVID-19 diagnosis using AI-ECG, the study [58] demonstrates the best practices for the analyzed case of diagnosis. This study stands out due to several compelling reasons, making it a prime example of effective application in this field. Attia Z.I.’s study utilizes an AI-ECG algorithm, which achieved a high negative predictive value (NPV) of 99.2%. This high NPV indicates that the algorithm is exceptionally effective in ruling out COVID-19 in patients. In a clinical setting, where the ability to rapidly and accurately exclude non-COVID cases is crucial, this high NPV is a significant advantage. Although the exact overall accuracy of the algorithm is not specified, the importance of the NPV in screening cannot be overstated. This makes the study’s findings highly relevant and practical for widespread clinical use.

The complexity and computational demands of more advanced networks, such as 3D CNNs and hybrid architectures, present notable challenges. For example, Sobahi N. [63] and Gomes J.C. [66] proposed models that, while potentially offering slight improvements in accuracy, require significantly higher computational resources. This makes them less feasible for rapid and widespread deployment, particularly in settings with limited resources. Moreover, these complex models often necessitate large datasets to generalize effectively, posing additional challenges in terms of data availability. The hybrid model proposed in [66] might be constrained by the availability of sufficiently large and diverse training data.

The implementation and maintenance of complex models in clinical practice can also be challenging. Simpler CNN models, like the one used in [64], are easier to integrate into existing healthcare infrastructure. They require less specialized knowledge to operate, making them more practical for everyday clinical use. The high NPV achieved by Attia Z.I.’s study has been validated in real-world scenarios, enhancing its reliability and suitability for clinical adoption. In contrast, other models might still be in the experimental stages or lack extensive validation, limiting their immediate applicability.

In summary, while more complex networks could potentially offer incremental improvements in accuracy, the balance of high NPV, ease of implementation, and practical validation demonstrates the best practices for COVID-19 diagnosis using AI-ECG. This study’s strengths highlight the importance of considering practical aspects such as computational requirements, data needs, and ease of integration into clinical practice when evaluating the effectiveness of AI models in healthcare.

#### 4.4.2. Diagnosis of Complications Associated with COVID-19

In the context of the COVID-19 pandemic, medical staff have been subjected to extreme stress and constant pressure, leading to an increased prevalence of burnout. A study by Gupta M.D. [70] estimated the prevalence of burnout in medical staff using the Mini Z scale. This study also developed an AI-based predictive model for detecting burnout. ECG data collected from exhausted individuals were used to create an AI model capable of predicting stress and burnout in HCWs during the COVID-19 pandemic.

Another important tool for diagnosing complications associated with COVID-19 infection is the detection of left ventricular failure. In 2020, Attia Z.I. et al. [71] proposed a tool to detect left ventricular failure in patients with COVID-19. The study emphasizes the importance of early identification of this complication to improve the prognosis of patients affected by COVID-19. Both studies highlight the crucial role of advanced technologies, such as AI and ECG analysis, in diagnosing and managing complications associated with COVID-19 infection, both among medical staff and patients.

Even though the studies do not refer to the same cause, we analyzed them from a methodological perspective. For the burnout study, the authors opted for an extra tree classifier due to its robustness in handling complex interactions between HRV features and its ability to perform well with the diverse data collected from a large sample of HCWs. For the AI ECG study, the authors chose a CNN because of its proven capability in identifying subtle patterns in ECG data, essential for accurately detecting left ventricular dysfunction in a clinical setting. The choice of these algorithms aligns with the specific requirements and challenges of the respective scenarios (Table 7).

In the realm of diagnosing complications arising from COVID-19, two significant studies have illustrated the potential of ML and AI in healthcare. The first study utilized an extra tree classifier to predict burnout among HCWs in India, revealing significant factors contributing to burnout and demonstrating the algorithm’s ability to accurately identify individuals at risk. This study underscores the importance of addressing mental health and working conditions to mitigate burnout in healthcare settings.

The second study applied a convolutional neural network (CNN) to ECG data, showcasing its effectiveness in detecting left ventricular dysfunction in COVID-19 patients. The high accuracy and non-invasive nature of this AI-enabled ECG analysis offer a promising tool for early diagnosis, potentially improving patient outcomes by enabling timely interventions.

Both studies, despite focusing on different complications, highlight the transformative role of advanced algorithms in diagnosing health issues. The extra tree classifier’s strength in handling complex interactions and the CNN’s capability in recognizing subtle patterns exemplify the tailored use of ML techniques to address specific medical challenges.

### 4.5. Challenges, Ethical and Societal Implications

The application of AI and ML in diagnosing COVID-19-related complications presents a range of challenges, ethical considerations, and societal implications that must be addressed to ensure responsible and effective use.

#### 4.5.1. Challenges

One of the primary challenges is ensuring the quality and availability of data. High-quality, representative datasets are crucial for training robust AI models, but data variability, incomplete records, and differences in data collection methods can hinder model performance. Integrating AI tools into everyday clinical settings requires seamless compatibility with existing healthcare systems, which can be technically challenging and resource intensive. Another significant challenge is the need for interpretable AI models. While high-performing AI models are essential, they must also be understood by clinicians to gain trust and facilitate clinical adoption. Generalizability is another key concern; AI models must be validated across diverse populations and healthcare settings to ensure their effectiveness and reliability. Models trained on specific datasets may not perform well in different clinical environments, highlighting the importance of extensive validation. Furthermore, navigating the regulatory landscape for AI in healthcare involves ensuring compliance with data privacy laws and obtaining necessary the approvals from health authorities, which can be a complex process.

#### 4.5.2. Ethical Considerations

The integration of AI in healthcare also raises important ethical issues. Protecting patient data from breaches and ensuring confidentiality is paramount, requiring robust data encryption and secure storage practices. AI models can perpetuate existing biases present in training data, leading to unfair treatment of certain patient groups. Developing strategies to detect and mitigate bias in AI systems is crucial. Ensuring transparency in AI decision-making processes and establishing clear accountability for outcomes is essential. Clinicians should be able to understand and trust AI-generated recommendations. Patients should be informed about the use of AI in their care and provide consent, with a clear understanding of how their data are being used and the potential implications.

#### 4.5.3. Societal Implications

The automation of certain tasks by AI may impact healthcare employment, necessitating strategies to retrain and upskill HCWs to adapt to new roles. Ensuring equitable access to AI technologies in healthcare is critical, as disparities in access can exacerbate existing healthcare inequalities. Building public trust in AI technologies requires transparent communication about their benefits, limitations, and the safeguards in place to protect patients. Developing comprehensive legal and regulatory frameworks to govern the use of AI in healthcare will ensure that ethical standards are maintained and that AI applications are safe and effective. Educating healthcare professionals and the public about AI technologies, their potential, and limitations will be essential for their acceptance and effective use. Encouraging international collaboration in AI research and development can lead to the creation of robust, universally applicable AI tools, fostering innovation and improving global health outcomes.

As AI continues to advance, addressing these challenges, ethical considerations, and societal implications will be essential for ensuring that these technologies are used responsibly and for the benefit of all patients. The integration of AI in healthcare promises significant improvements in diagnostic accuracy and patient outcomes, but it must be approached with careful consideration of its broader impacts.

## 5. Discussions on Future Directions and Challenges

As the integration of AI and ML in healthcare continues to advance, several emerging trends and challenges must be addressed to ensure their effective and ethical deployment. This chapter explores these future directions and highlights the technical, ethical, and societal implications that come with the adoption of AI in healthcare, particularly in the context of cardiovascular disease management and the ongoing COVID-19 pandemic.

### 5.1. Emerging Trends, Technical Challenges, and Research Needs

When considering the current developments in the field, it is evident that AI and ML technologies are rapidly evolving, presenting new opportunities for enhancing healthcare delivery. One notable trend is the increased use of DL models for complex data analysis. For instance, CNNs and RNNs have shown high accuracy in diagnosing conditions such as arrhythmias and myocardial infarction from ECG data. Furthermore, the development of hybrid models that combine the strengths of different algorithms, such as combining CNNs with SVMs or utilizing ensemble learning approaches can enhance model robustness and interpretability in handling the diverse and complex nature of healthcare data.

However, despite these advancements, there remain several technical challenges that must be addressed to fully realize the potential of AI in healthcare. One significant issue is the necessity of high-quality, representative datasets for training robust AI models. Data variability, incomplete records, and differences in data collection methods can significantly hinder model performance. Additionally, the integration of AI tools into everyday clinical settings also requires seamless compatibility with existing healthcare systems, which can be technically challenging and resource intensive. Interpretability also remains a critical factor, as AI models must be understood by clinicians to gain their trust and facilitate broader clinical adoption.

Another important consideration is the need for extensive validation of AI models across diverse populations and healthcare settings. The generalizability of these models is a key concern, as those trained on specific datasets may not perform adequately in different clinical environments. Therefore, validation efforts must ensure the effectiveness and reliability of AI applications in various contexts.

Furthermore, addressing the readiness of healthcare systems to adopt AI technologies is crucial. Implementing AI systems that continuously learn and adapt from new data requires substantial investment in infrastructure, training, and change management. These foundational elements are essential for the successful integration and utilization of AI tools within healthcare facilities. Continued research, collaboration, and investment in healthcare infrastructure are essential to harnessing the full potential of AI and ensuring its benefits are realized across diverse populations and healthcare settings.

### 5.2. Healthcare System Readiness

Implementing AI systems that can continuously learn and adapt from new data will enhance their accuracy and relevance, ensuring they remain up to date with the latest medical knowledge. However, this requires substantial investment in infrastructure, training, and change management to ensure the successful integration and utilization of AI tools.

Healthcare facilities must invest in robust IT infrastructure to support the deployment and maintenance of AI systems. This includes high-performance computing resources, secure data storage solutions, and reliable network capabilities. Without these foundational elements, the potential of AI cannot be fully realized.

Educating healthcare professionals about AI technologies is crucial for their acceptance and effective use. This includes training clinicians to interpret AI-generated insights and integrate them into clinical decision-making processes. Continuous professional development programs can help HCWs stay abreast of advancements in AI and ML, fostering a culture of innovation and adaptability.

Successfully integrating AI into healthcare requires careful change management strategies. This involves engaging stakeholders at all levels, from top management to frontline staff, to build consensus and address concerns about the new technology. Clear communication about the benefits and limitations of AI can help alleviate resistance and foster a collaborative environment. AI tools must be compatible with existing electronic health records (EHR) and other healthcare information systems. Ensuring interoperability can facilitate seamless data exchange and integration, allowing AI systems to function effectively within the broader healthcare ecosystem. Standardizing data formats and protocols can further support this integration.

AI systems must comply with data privacy laws, such as HIPAA in the United States, and obtain the necessary approvals from health authorities. Ensuring compliance with these regulations is critical to maintaining patient trust and safeguarding sensitive health information.

### 5.3. Collaboration and Innovation

Encouraging international collaboration in AI research and development can lead to the creation of robust, universally applicable AI tools.

Establishing global research networks can facilitate the sharing of knowledge, resources, and best practices. By collaborating across borders, researchers can pool diverse datasets, which can improve the generalizability of AI models. International collaborations can also accelerate the development of new algorithms and applications, leveraging the expertise of a diverse range of specialists.

Partnerships between public institutions and private companies can drive innovation in AI healthcare technologies. Private sector involvement can provide the necessary funding and technological expertise, while public institutions can offer clinical insights and access to patient data. These collaborations can lead to the rapid translation of research findings into practical, scalable solutions.

Developing comprehensive ethical frameworks is essential to guide the responsible use of AI in healthcare. International cooperation can help establish standards for data privacy, informed consent, and bias mitigation. These frameworks can ensure that AI technologies are deployed in a manner that respects patient rights and promotes equitable access to healthcare.

Creating innovation hubs and incubators can support the development and testing of AI healthcare solutions. These hubs can provide startups and researchers with access to funding, mentorship, and state-of-the-art facilities. By fostering a collaborative environment, innovation hubs can accelerate the commercialization of new AI technologies and their integration into healthcare systems.

Advocating for supportive policies and regulatory frameworks is crucial for the advancement of AI in healthcare. Policymakers must be informed about the potential benefits and risks of AI technologies to create regulations that promote innovation while safeguarding public interests. Collaborative advocacy efforts can influence policy decisions and ensure that AI development aligns with societal values. Engaging the public in discussions about AI in healthcare can build trust and acceptance. Transparent communication about the potential benefits, limitations, and safeguards associated with AI technologies is essential. Public education campaigns can help demystify AI and address common misconceptions, fostering a more informed and supportive community.

However, addressing the technical, ethical, and societal challenges is essential for the responsible and effective use of these technologies. Continued research, collaboration, and investment in healthcare infrastructure are crucial to harnessing the full potential of AI and ensuring its benefits are realized across diverse populations and healthcare settings.

## 6. Conclusions

This comprehensive review has explored the transformative potential of AI and ML in healthcare, particularly using AI-ECGs for managing cardiovascular diseases, COVID-19-related complications, and cardio-oncology-associated complications. By examining various AI methodologies, their applications, and the challenges they present, we underscore the significant improvements these technologies can bring to diagnostic accuracy and patient outcomes.

The primary ML techniques discussed include convolutional neural networks (CNNs), which excel in recognizing spatial patterns within ECG data, making them highly effective for arrhythmia detection and myocardial infarction diagnosis. DL demonstrates high accuracy in handling complex, multi-dimensional ECG data, which is crucial for conditions such as PH and mortality prediction. K-Nearest Neighbors (KNN) is suitable for small-scale studies and simpler applications, such as hypertension detection. The extra tree classifier is robust in handling complex interactions and is applied in predicting burnout among healthcare workers during the COVID-19 pandemic.

The integration of AI with ECGs significantly enhances diagnostic accuracy and early disease detection, leading to improved patient management and outcomes. AI-ECG provides precision by identifying subtle patterns and anomalies in ECG data that are often missed by traditional methods. It enables quick, non-invasive, and real-time diagnosis and monitoring, which are particularly critical during a pandemic, like it was in the COVID-19 pandemic. Additionally, AI-ECG facilitates the analysis of extensive digital ECG data, supporting large-scale health initiatives and personalized medicine.

For arrhythmias, CNNs are highly effective for detecting and predicting conditions such as atrial fibrillation and ventricular tachyarrhythmias. In the case of HF, CNNs excel in predicting HF by analyzing ECG signals, and DL models are also effective due to their ability to handle temporal sequences and complex data relationships. For PH, DL models are highly recommended as they can process and analyze complex ECG data with high accuracy. When diagnosing myocardial infarction and AMI, DL models and CNNs are recommended for their high accuracy and reliability. In managing COVID-19 complications, AI-ECG systems using CNNs, and DL models can predict the severity of COVID-19 and associated cardiovascular complications. For detecting hypertension, KNN models provide a practical tool for hospital diagnoses and continuous monitoring.

The field of cardio-oncology is also benefiting from AI advancements. CNNs and DL models have demonstrated proficiency in managing chemotherapy-induced cardiotoxicity and detecting cardiac masses in high-risk groups, such as childhood cancer survivors. These models can capture subtle patterns in ECG data, enabling early intervention and improving long-term outcomes for cancer patients who are at risk of developing cardiovascular diseases as a result of their treatment.

The development of hybrid models combining different AI algorithms is expected to enhance robustness and accuracy. AI systems capable of continuous real-time patient monitoring and immediate interventions are also on the horizon. The integration of various data sources, including genetic information, lifestyle factors, and clinical history, will drive the trend towards personalized medicine. Additionally, encouraging global research initiatives will help develop universally applicable AI tools.

The integration of AI and ML in cardiology, particularly through AI-ECG, offers transformative improvements in the prediction, diagnosis, and management of cardiovascular diseases. Algorithms such as CNNs and DL models are especially effective in handling complex and high-dimensional medical data. These models excel in capturing intricate patterns within ECG data, leading to more accurate and timely diagnoses. For example, DL models have shown high accuracy in diagnosing arrhythmias, myocardial infarction, and mitral regurgitation, significantly improving early intervention and patient outcomes. CNNs have demonstrated their proficiency in predicting and diagnosing cardiomyopathy, particularly in high-risk groups such as childhood cancer survivors. The ability of these advanced algorithms to process and analyze large datasets allows them to identify subtle patterns and anomalies that traditional methods might miss.

The research and application of AI in cardiology highlight the potential for these technologies to further advance precision medicine, improve patient outcomes, and enhance clinical decision-making. As research continues to evolve, we can expect AI to play an increasingly integral role in the management and treatment of cardiovascular diseases, offering new opportunities for early diagnosis, risk prediction, and personalized care. The ongoing development and refinement of AI algorithms will likely lead to even more sophisticated tools and techniques, driving further improvements in cardiovascular healthcare.

This work provides a detailed examination of AI and ML technologies applied to heart diseases and COVID-19-related complications, with a specific focus on AI-ECGs. By exploring various AI methodologies and their impact on diagnosing and managing cardiovascular diseases, this review underscores the transformative potential of AI in healthcare. Addressing the challenges and leveraging future trends will be crucial for harnessing AI’s full potential, ultimately improving patient outcomes and advancing the field of cardiovascular medicine. This comprehensive review serves as both an insightful guide and a call to action for further research and collaboration in the integration of AI in cardiology.

## Figures and Tables

**Table 1:** Overview of key ML algorithms [16] and their use cases.

Algorithm	Key Characteristics	Use Cases	Advantages	Disadvantages
Convolutional Neural Networks (CNNs)	Specialized for processing grid-like data such as images, extensively used in medical image analysis	Predicting and diagnosing arrhythmias, heart failure, myocardial infarction, and COVID-19 complications	High accuracy in image and pattern recognition, effective for complex data	Require large datasets, computationally intensive
Recurrent Neural Networks (RNNs)	Effective for sequential data like time series or heart rate data, identifies patterns over time	Heart failure risk assessment, time series analysis	Capture temporal patterns, suitable for sequential data	Computationally intensive, require large datasets.
Autoencoders	Used for unsupervised learning tasks, anomaly detection in ECG data	Anomaly detection in ECG data	Effective for unsupervised learning, detect anomalies	Limited to unsupervised tasks, may not generalize well
Dense Neural Networks (DNNs)	Traditional neural networks with full connectivity, versatile for various predictive tasks	Various predictive tasks in medical data analysis	Versatile, used for a wide range of predictive tasks	Computationally intensive, require large datasets
K-Nearest Neighbors (KNN)	Simple, non-parametric, classifies based on the majority class among nearest neighbors	Hypertension detection, hospital diagnoses, continuous monitoring	Simple, easy to implement, effective for small datasets	Less effective for large datasets, may not handle complex relationships well
Extra Tree Classifier	Robust feature extraction and prediction, handles complex interactions	Predicting burnout among healthcare workers	Handles complex interactions, robust prediction	Computationally intensive, requires large datasets
Support Vector Machines (SVMs)	Used for classification and regression, combines multiple predictors for enhanced accuracy	Enhancing diagnostic accuracy in combination with CNNs	High accuracy, interpretable, robust to overfitting	Require careful tuning of parameters, may not handle temporal data effectively
Ensemble Learning Random Forest, XGBoost	Combines strengths of different algorithms, robust to overfitting, handles missing data well	Robust prediction models for cardiovascular diseases	Handles missing data well, robust predictions	Computationally intensive, requires large datasets
Hybrid Models	Combine multiple algorithms, enhance robustness and interpretability	Combining CNNs with SVMs or ensemble methods for improved diagnostics	Combine strengths of various models, highly robust and interpretable	Complex implementation, require large datasets

**Table 2:** Applications and findings of ML methods in risk prediction of heart affections.

Affections	Method	First Author	Brief Description of the Paper Topic
Arrythmias	ML	Baqal O. (2024) [17]	Identifying AF in normal sinus rhythm: AI-ECG can detect high-risk ECG features invisible to the human eye.
		Choi J. (2024) [18]	Effectiveness of AI in predicting AF: AI improves patient prognosis through prompt interventions.
		Pipilas D. (2023) [19]	Prioritization of preventive interventions: AI prioritizes individuals at high risk of AF for preventive measures.
		Raghunath S. (2023) [20]	Predicting new-onset atrial AF: AI helps in predicting AF from sinus rhythm ECGs.
	CNN	Raghunath A. (2023) [21]	Diagnosis of paroxysmal AF: AI predicts AF from sinus rhythm ECGs, often escaping early diagnosis.
		Baj G. (2023) [22]	Machine learning methods for AF prediction: The development of ECG-based prediction models is booming.
		Jiang, J. (2023) [23]	Catheter ablation and AF recurrence: AI-ECG predicts the risk of recurrence in paroxysmal AF post-ablation.
		Bai Y. (2021) [24]	Predicting AF recurrence post catheter ablation: AI-ECG models for predicting AF recurrence post-ablation.
	DL	Raghunath S. (2021) [25]	Prediction of incipient AF with deep learning: DL predicts AF using 12-lead ECG in patients with no known history of AF.
		Holmstrom L. (2024) [26]	Assessment of sudden cardiac death risk: DL model identifies cases at risk of sudden cardiac death (SCD).
Heart failure	CNN	Grun D. (2021) [27]	Predicting heart failure using ECG signals: AI demonstrates high accuracy in predicting HF with ECG.
		Akbilgic O. (2021) [28]	Early intervention in reducing morbidity and mortality: AI aids in HF risk prediction through ECG analysis.
Pulmonary hypertension	DL	Kwon J.M. (2020) [29]	Early diagnosis of PH: AI algorithm demonstrates high accuracy in predicting PH using both 12-lead and single-lead ECGs.
Mortality prediction	DL	Tsai D.J. (2023) [30]	DL-based survival model: Establishes critical value of ECG for CVD management and predicts SCD.
		Baek Y.S. (2023) [31]	Prediction of mortality: DL model estimates cardiac age and predicts mortality using 12-lead ECG.
	CNN	Shiraishi Y. (2023) [32]	Evaluating predictive models for SCD: ECG-based AI enhances prediction of sudden cardiac death (SCD).
		Liu C.M. (2022) [33]	Risk of cardiovascular mortality: AI model identifies pulmonary hypertension and predicts cardiovascular mortality.
Cardiomyopathy	CNN	Güntürkün F. (2021) [34]	Predicting late-onset cardiomyopathy: AI identifies childhood cancer survivors at risk of cardiomyopathy.
		Siontis K.C (2021) [2]	Detecting hypertrophic cardiomyopathy (HCM): DL model accurately diagnoses HCM using 12-lead ECG.
		Ko W.Y. (2020) [35]	High performance in HCM detection: AI complements traditional diagnostic methods, enhancing speed and accuracy.
Chemotherapy-induced cardiotoxicity	CNN	Jacobs JEJ (2024) [36]	AI-ECG detects newly abnormal left ventricular ejection fraction after anthracycline therapy.
	DL	Yagi R. (2024) [37]	AI predicts cardiotoxicity from baseline ECGs in chemotherapy patients.
		Halasz G. (2024) [38]	AI-assisted ECG provides accessible diagnosis of cancer therapy-related cardiac dysfunction.
	ML	Güntürkün F. (2021) [34]	AI predicts late-onset cardiomyopathy among childhood cancer survivors.

**Table 3:** Applications and findings of ML methods in diagnosis of heart disease.

Affections	Method	First Author	Brief Description of the Paper Topic
Arrythmias	DL	Baek Y.S. (2023) [39]	Identifying AF: DL-based algorithm shows high accuracy in detecting AF from 12-lead ECG during normal sinus rhythm.
		Baek Y.S. (2021) [40]	High accuracy in AF detection: DL model effectively identifies AF in clinical settings.
	DNN	Sabut S. (2021) [41]	Detection of ventricular arrhythmia: AI accurately detects ventricular tachyarrhythmia conditions.
		Bos J.M. (2021) [42]	Early detection of congenital long QT syndrome: AI differentiates patients with electrocardiographically occult long QT syndrome.
Mitral regurgitation	DL	Kwon J.M. (2020) [43]	Detecting mitral regurgitation: AI algorithm shows promising results using 12-lead and single-lead ECGs.
Hypertension	KNN	Soh D. C.K. (2020) [44]	Detection of hypertension: Computational intelligence tool uses ECG signals for HPT detection and identification of masked hypertension.
Pulmonary embolism	DNN	Valente S.B. (2023) [45]	Diagnosis of acute pulmonary embolism: DL model shows high specificity in diagnosing PE using 12-lead ECG.
Myocardial infarction	DL	Liu W.C. (2021) [46]	Detecting myocardial infarction: DL model aids in timely diagnostic decision-making using 12-lead ECG.
		Liu W.C. (2021) [47]	Diagnosis of acute myocardial infarction: AI algorithm successfully detects MI with both 12-lead and 6-lead ECG.
	CNN	Cho Y. (2020) [48]	Detecting myocardial infarction: AI model demonstrates exceptional diagnostic capability for STEMI.
		Zhao Y. (2020) [49]	Early detection of STEMI: AI-S trial shows significant improvement in time to treatment in AMI cases.
Cardiac Masses in Cardio- Oncology	CNN	Oikonomou EK. (2024) [50]	Stratification of cancer-related cardiac dysfunction using ECG imaging.
	DL	Martinez DS. (2022) [51]	AI in cardio-oncology: Enhancing ECG-based diagnosis of cardiac masses.

**Table 4:** Analysis of studies on AI prediction of COVID-19 complications.

Study	Algorithm	Strengths	Weaknesses	Accuracy	Clinical Relevance	Data Source	Key Findings
Baek Y. (2023) [54]	CNN	High accuracy in image recognition, capable of detecting subtle patterns in ECG	Requires large dataset, computationally intensive	High	High, as it directly processes ECG	Multi institutional dataset	CNN accurately predicts cardiovascular complications from ECGs, demonstrating its utility in clinical settings
Nawaz M. (2022) [52]	Ensemble Learning (Random Forest, XGBoost)	Handles missing data well, interpretable, robust to overfitting	May not capture temporal data effectively	Moderate to High	Moderate, focuses on combining various predictors	Hospital records, ECGs, clinical data	Ensemble models performed well with integrated clinical and ECG data, highlighting their robustness
Sridhar A.R. (2022) [7]	CNN-LSTM	Captures temporal patterns in ECG, good for sequential data	Computationally intensive, requires large dataset	Moderate	High, predicts mortality and MACE from ECGs	Multi institutional dataset	CNN-LSTM had limited predictive power alone, suggesting need for mixed-input models for improved accuracy
Gupta M.D. (2021) [55]	Extra Tree Classifier	Good feature importance analysis, interpretable, robust	Lower sensitivity compared to DL methods	Moderate	High, predicts burnout and stress in HCWs using HRV	Multicenter dataset	Extra tree classifier effectively distinguished between burnout and non-burnout cases with HRV data, Demonstrating its utility in stress prediction

**Table 5:** Analysis of studies on AI prediction of COVID-19 mortality risk.

Study	Algorithm	Strengths	Weaknesses	Accuracy	Clinical Relevance	Data Source	Key Findings
Sbrollini et al. (2024) [56]	AdvRS&LP with LIME	High interpretability; robust feature extraction; high AUC	Limited dataset size; Complexity of the model	AUC ^1^: 84.31%	High: Easily interpretable and clinically relevant	COVIDSQUARED project	Identified ECG and VCG patterns related to COVID-19 mortality with high accuracy and interpretability.
Van de Leur et al. (2022) [57]	Logistic Regression, LASSO, DNN	Comparison of multiple models; external validation; automated approach	Decrease in performance during external validation; less interpretable	AUC: 77% (DNN)	Moderate: Automated but less interpretable	CAPACITY-COVID registry	Showed ECG-based models can predict COVID-19 mortality; DNN performed similarly to human annotation but with limited added value above age and sex.

^1^ Area under the curve. It is a metric used to measure the performance of a binary classification model. The AUC represents the degree or measure of separability and indicates how well the model can distinguish between classes. A higher AUC value indicates better model performance.

**Table 6:** Analysis of studies on AI diagnosis of COVID-19 using ECG.

Study	Algorithm	Strengths	Weaknesses	Accuracy	Clinical Relevance	Data Source	Key Findings
Sakr A.S. (2023) [58]	CNN + RNN	Good balance of precision and sensitivity	Slightly lower accuracy compared to others	96.0 (Binary)	Useful for real-time diagnosis	Public ECG dataset	Combines temporal and spatial features effectively, achieving robust diagnostic performance
Agrawal A. (2022) [59]	CNN + SVM	High accuracy and specificity	Requires careful tuning of SVM parameters	97.8 (Binary) ^1^	Suitable for clinical implementation	Public ECG dataset	SVM enhances the classification capability of CNN, resulting in high diagnostic accuracy
Irmak E. (2022) [60]	CNN + Data Augmentation	Reduces overfitting ^2^, enhances model robustness	Lower accuracy compared to other models	81.8 (Multiclass) ^3^	Useful for improving model generalization	Public ECG dataset	Data augmentation improves robustness but may not significantly enhance accuracy in complex multiclass classification tasks
Nguyen T. (2022) [61]	CNN + Transfer Learning	High specificity and precision	Moderate sensitivity	97.62 (Binary)	Effective for COVID-19 diagnosis	Public ECG dataset	Demonstrates the effectiveness of transfer learning in improving ECG-based COVID-19 diagnosis accuracy
Rahman T. (2022) [62]	Hexaxial ^4^ Feature Mapping + CNN	High sensitivity	Moderate specificity	96.2 (Binary)	Effective for initial screening	Public ECG dataset	Hexaxial feature mapping improves sensitivity, making it effective for initial screening of COVID-19
Sobahi N. (2022) [63]	EfficientNet ^5^	Efficient model, low computational cost	Lower accuracy compared to more complex models	81.8 (Multiclass)	Suitable for resource-limited settings	Public ECG dataset	EfficientNet offers a balance between efficiency and accuracy, suitable for quick screening in resource-limited settings
Attia Z.I. (2021) [64]	Hybrid CNN + Traditional ML	High accuracy, combines multiple models, feature selection	Complex, requires multiple models	98.2 (Binary), 91.6 (Multiclass)	Potential alternative diagnostic tool	Public ECG dataset	High accuracy in diagnosing COVID-19 using ECG, demonstrates potential as an alternative diagnostic tool
Ozdemir M.A. (2021) [65]	EfficientNet	Efficient model, low computational cost	Lower accuracy compared to more complex models	81.8 (Multiclass)	Suitable for resource-limited settings	Public ECG dataset	EfficientNet offers a balance between efficiency and accuracy, suitable for quick screening in resource-limited settings
Gomes J.C. (2023) [66]	Multi-Branch Fusion Network ^6^	High accuracy, handles multiple features	Complex architecture	98.0 (Binary)	Highly reliable for clinical diagnosis	Public ECG dataset	Multi-branch fusion network effectively combines multiple feature sets, resulting in high diagnostic accuracy
Bassiouni M.M. (2022) [67]	CNN + LSTM ^7^	Handles temporal dependencies well	Requires large amounts of data for training	97.0 (Binary)	Effective for dynamic ECG signal analysis	Public ECG dataset	LSTM effectively captures temporal features, improving COVID-19 diagnosis from ECG data
Prashant K. (2022) [68]	CNN + RF	High accuracy, combines ensemble learning	Computationally intensive	98.0 (Binary)	Reliable for clinical use	Public ECG dataset	Random Forest improves robustness and accuracy of CNN in diagnosing COVID-19 from ECG
Attallah O. (2022) [69]	Hybrid CNN + Traditional ML	High accuracy, combines multiple models, feature selection	Complex, requires multiple models	98.2 (Binary), 91.6 (Multiclass)	Potential alternative diagnostic tool	Public ECG dataset	High accuracy in diagnosing COVID-19 using ECG, demonstrates potential as an alternative diagnostic tool

^1^ Refers to a classification task where there are only two possible classes or outcomes. In the context of these studies, binary classification typically involves determining whether a patient is COVID-19 positive or negative based on ECG data. ^2^ This occurs when a ML model learns the details and noise in the training data to the extent that it negatively impacts the performance of the model on new data. Overfitting means the model performs well on training data but poorly on unseen data. ^3^ Refers to a classification task where there are more than two classes or outcomes. In these studies, multiclass classification could involve categorizing ECG data into multiple health conditions, not just the presence or absence of COVID-19. ^4^ Relates to the hexaxial reference system, which is a method used to interpret the electrical activity of the heart in the frontal plane of the body. It helps in the analysis of ECG signals by providing a comprehensive view of heart activity from multiple angles. ^5^ A family of convolutional neural networks that are optimized for both accuracy and efficiency. Efficient net models are known for their ability to achieve high accuracy with fewer computational resources, making them suitable for deployment in environments with limited computational power. ^6^ A type of neural network architecture that combines multiple branches, each responsible for learning different features from the input data. This approach allows the network to capture a diverse set of features, improving its overall performance on complex tasks. ^7^ Long short-term memory (LSTM): A type of RNN that is capable of learning long-term dependencies. LSTMs are particularly effective for tasks where the sequence of data points matters, such as analyzing time-series data like ECG signals.

**Table 7:** Comparative analysis of AI models for burnout and left ventricular dysfunction detection in COVID-19 contexts.

Study	Algorithm	Strengths	Weaknesses	Accuracy	Clinical Relevance	Data Source	Key Findings
Gupta, M. D. (2021) [70]	Extra tree classifier	Large sample size (1615 participants), use of validated Mini-Z 1.0 Survey ^1^, comprehensive analysis of HRV features, multicentric study	Cross-sectional study, self-reported burnout measure, limited to Indian HCWs ^2^	Sensitivity: 84%, AUC: 84%, Accuracy: 77%	Highlights the need for mental health support and better working conditions for HCWs, Potential for ML models to predict burnout and inform interventions	HCWs from four academic centers in India	Burnout rate: 16.2% overall, higher in second-line workers (20.5%), lower HRV in burned-out HCWs, significant factors: stress, job dissatisfaction, chaotic environment, mental impact of COVID-19
Attia Z. I. (2020) [71]	AI-enabled ECG analysis using a CNN	High accuracy (AUC 95%), rapid, noninvasive screening, FDA ^3^ emergency use authorization, real-world clinical data from Mayo Clinic	Small sample size (27 patients), retrospective study, lack of routine rhythm surveillance and other clinical markers	AUC: 95%	Provides a tool for early detection of cardiac dysfunction in COVID-19 patients, minimizes need for echocardiography, reducing healthcare staff exposure	COVID-19 patients at Mayo Clinic	AI ECG accurately detected EF ^4^ ≤40%, one patient with myocarditis showed rapid clinical decline detected by AI ECG, AI ECG effective in detecting pre-existing and new LV ^5^ dysfunction

^1^ A validated survey instrument used to measure burnout and job satisfaction among healthcare workers. ^2^ Healthcare Workers. ^3^ U.S. Food and Drug Administration. ^4^ Ejection fraction, a measurement of the percentage of blood leaving the heart each time it contracts. ^5^ Left ventricle, the chamber of the heart responsible for pumping oxygenated blood to tissues all over the body.

## Data Availability

Not applicable.

## Abbreviations

AdvRS&LP	advanced repeated structuring and learning procedures
AF	atrial fibrillation
AI	artificial intelligence
AI-ECG	AI-enhanced electrocardiograms
AMI	acute myocardial infarction
AUC	area under the curve
CNN	convolutional neural networks
COVID-19	coronavirus disease 2019
DNN	dense neural networks
DL	deep learning
ECG	electrocardiography
EfficientNet	a family of convolutional neural networks optimized for accuracy and efficiency
EF	ejection fraction
FDA	U.S. Food and Drug Administration
HBP	high blood pressure
HCWs	healthcare workers
HCM	hypertrophic cardiomyopathy
HRV	heart rate variability
KNN	K-Nearest Neighbors
LSTM	long short-term memory
LV	left ventricle
MHPT	masked hypertension
MI	myocardial infarction
ML	machine learning
NLP	natural language processing
NN	neural networks
PE	pulmonary embolism
PH	pulmonary hypertension
RNN	recurrent neural networks
SCD	sudden cardiac death
STEMI	ST-segment elevation acute myocardial infarction
VCG	vectorcardiography
